# Supplementary material for: Chromothripsis during telomere crisis is independent of NHEJ, and consistent with a replicative origin
Source: Genome Res. 2019 May;29(5):737–49. doi: 10.1101/gr.240705.118 (PMC6499312; doi:10.1101/gr.240705.118)
Supplement: Supplemental Material [file supp_gr.240705.118_Supplemental_file_1.zip › contigs/annotated_contigs/DB56/contig.2.DB56_length_264_mean_cov_10.0113636364.docx]

**DB56_length_264_mean_cov_10.0113636364**

```
AAAAATACAAAATTTAGTTGGGCATGGAGGCATGCACTTGTAATCTCAGCTACTCAAGAGGTTAAGGCACAAGAATCACTTGAACCTGG
 >chr3:61538968-61539187 - E=5e-110 p=0e+00
GAGGCAGAGGTTGCAGTGAGCTGAGATGGTGTGACTGCACTCCACCTGGGTGACAGACTAAGACTTTGTCTCAAAAAGAGAGAAAAAAA

CAAAAAAAAAACTCTGAAAGAGCACAAAATGACAAACAAA|AG|AAAAAAAAAAAAAAAAAAAAAAAAAAAAGAACACAAAAAAAAAA                                           >chr19:21451932-21451976 + E=4e-12
```
